# E-cigarettes: methodological and ideological issues and research priorities

**DOI:** 10.1186/s12916-014-0264-5

**Published:** 2015-02-16

**Authors:** Jean-François Etter

**Affiliations:** Jean-François Etter, Institute of Global Health, University of Geneva, CMU, case postale, CH-1211 Geneva 4, Switzerland

**Keywords:** Tobacco use disorder, Electronic nicotine delivery devices (ENDS), Electronic cigarette, E-cigarette, Nicotine, Smoking, Tobacco

## Abstract

Cigarette combustion, rather than either tobacco or nicotine, is the cause of a public health disaster. Fortunately, several new technologies that vaporize nicotine or tobacco, and may make cigarettes obsolete, have recently appeared. Research priorities include the effects of vaporizers on smoking cessation and initiation, their safety and toxicity, use by non-smokers, dual use of vaporizers and cigarettes, passive vaping, renormalization of smoking, and the development of messages that effectively communicate the continuum of risk for tobacco and nicotine products. A major difficulty is that we are chasing a moving target. New products constantly appear, and research results are often obsolete by the time they are published. Vaporizers do not need to be safe, only safer than cigarettes. However, harm reduction principles are often misunderstood or rejected. In the context of a fierce ideological debate, and major investments by the tobacco industry, it is crucial that independent researchers provide regulators and the public with evidence-based guidance. The methodological and ideological hurdles on this path are discussed in this commentary.

## Background

The combustion of cigarettes, rather than either tobacco or nicotine, is the cause of a public health disaster. The cigarette-rolling machine, an innovation of the 19th century, bears much of the responsibility for this disaster. However, a series of 21st century innovations have the potential to revert the statistics back to the very low levels of tobacco-related mortality that existed before the advent of manufactured cigarettes, when tobacco was used mainly in non-combustible forms. Recent innovations include electronic cigarettes, vaporizers that heat tobacco but do not burn it, products similar to asthma inhalers that produce a nicotine aerosol, products that use a chemical reaction (pyruvate) to vaporize nicotine and products that use a flow of hot air to vaporize tobacco [1-4]. Given the profitability of this market, there is little doubt that other types of vaporizers will soon appear.

Scientists and regulators often react in a confused way to these disruptive technologies. The debate is highly ideological, and arguments are often misleading and even dishonest [5]. Negative reports on e-cigarettes tend to receive much media coverage, and, as a result, the proportion of smokers who think that e-cigarettes are more dangerous than combustible cigarettes is increasing [6]. In this context, it is crucial to provide regulators, clinicians, journalists and consumers with sound, evidence-based responses to their questions. However, there are hurdles on this path, both methodological and ideological.

## Research priorities

The impact of e-cigarettes and vaporizers on public health is the product of the damage caused or prevented by such products, multiplied by the number of people who adopt these products and stop using combustible cigarettes. From a regulatory point of view, the objective should be to minimize the harm to the population caused by all nicotine and tobacco products, including combustible cigarettes. Researchers should provide evidence to help regulators write reasonable regulations that take into account a continuum of risk for nicotine and tobacco products, are based on a principle of proportionality and do not stifle innovation. Research priorities include the effects of vaporizers on smoking cessation and initiation, safety and toxicity, use by non-smokers, dual use of vaporizers and cigarettes, use in public places (exposure to passive vaping and renormalization of smoking), flavors (toxicity and behavioral effects), nicotine (addictiveness, toxicity, risk perception) and the development of messages that effectively communicate the continuum of risk.

## Methodological issues

A major difficulty is that we are chasing a moving target. New products appear all the time, and research findings are often obsolete by the time they are published.

Most published studies on e-cigarettes are relatively short-term, but it is crucial to assess the long-term effects of these products on health and behavior. This will be costly and take years, and long-term studies will be outdated by the time they are published.

One important question is to assess whether e-cigarettes are a gateway to smoking or nicotine dependence in young non-smokers. We know from research on illicit drugs (is cannabis a gateway to heroin?) that proving gateway effects requires methodologically sophisticated studies [7]. For e-cigarettes, all published studies to date that address gateway effects fall short of these methodological requirements [4,8].

Assessing the effects of passive exposure to e-cigarette vapors is also politically relevant. However, given the very low levels of risk involved (probably orders of magnitude lower than for cigarette smoke), any health effect will be very hard to detect.

## Ideological bias

There is a continuum of risk for nicotine-containing products [9,10]. Harm reduction is about the lesser of two evils: vaporizers need to be safer than cigarettes, but not necessarily safe. Failure to admit this leads to negative attitudes towards reduced-risk products and to regulations that apply the same restrictions to all products. This is damaging to public health, as it hampers alternatives to combustible cigarettes. In many countries, nicotine vaporizers and smokeless tobacco cannot currently be advertised as reduced-risk products. Laws that prevent truthful communication about this continuum of risk prevent adoption of less harmful alternatives to combustible cigarettes.

The debate is loud but lacks robustness, and there is often an ideological bias against, and a lack of understanding of, harm reduction principles. There is also a willingness of the press and some scientists to emphasize the negative effects of e-cigarettes [5]. In particular, press releases issued by scientists or by their institutions sometimes do not reflect research findings [11]. This could be prevented by submitting press releases to the same peer-review process as that for scientific articles. The public has the right to an objective assessment of the situation, and to appropriate guidance, but at present this is not what it gets from many scientific articles, press reports and institutions [5,11,12].

## Conflicts of interest

Most e-cigarette manufacturers have shown little interest in conducting or supporting peer-reviewed research. Much of the research is, therefore, conducted by independent researchers, but conflicts of interest are nevertheless present [1]. In contrast, research on other types of vaporizers (for example, heated tobacco products) is mainly conducted by the tobacco industry. For instance, Philip Morris International invested $2bn in research and development efforts for their four new vaporizing technologies [13]. To counterbalance this enormous investment, other funding sources (governments, foundations, crowdfunding) are needed to support independent researchers. A small tax (a few cents per unit) could be imposed on vaporizers to support independent research and education, but otherwise these products should be given a tax incentive.

The tobacco industry will probably soon dominate the nicotine/tobacco vaporizers market, not least because excessive regulation will make it too costly for smaller players to survive in a highly regulated environment. The tobacco industry will then be in a position to stifle this market, if it ends up being less profitable than tobacco cigarettes. Because of the dominant position of the tobacco industry, researchers and their institutions have little choice but to (reluctantly) reconsider their attitudes towards this industry. This is one of the thorniest issues in this field. The disclosure of conflicts of interest and registration of studies in open registries are necessary but insufficient first steps. An open debate, involving all stakeholders, is needed on this issue. Transparency and an appropriate approach to the management of conflicts of interest are necessary to preserve the integrity of research and public trust.

## Conclusions

A window of opportunity is now open but will soon close. In the United States, the Food and Drug Administration is developing regulations that will apply to e-cigarettes and vaporizers, and European Union Member States are now transposing the Tobacco Products Directive into national laws. Once these regulations are in place, they will be very difficult to change. However, because harm reduction strategies are often misunderstood or rejected [14], there is a risk that e-cigarettes and vaporizers will be excessively regulated. Regulators must consider the unintended consequences of excessive regulation, and should be held accountable for any such consequences. Given that e-cigarettes and vaporizers are already much safer than combustible cigarettes, any benefit of regulations will be small, whereas the unintended consequences can have a large negative impact. Unfortunately, current proposals for regulation are often worse than the status quo. It is sad that this is happening with the help of some public health professionals, scientists and elected representatives of the people.
